# Supplementary figures and images for: Metabolomic profiling of oxalate-degrading probiotic Lactobacillus acidophilus and Lactobacillus gasseri
Source: PLoS One. 2019 Sep 23;14(9):e0222393. doi: 10.1371/journal.pone.0222393 (PMC6756784; doi:10.1371/journal.pone.0222393)

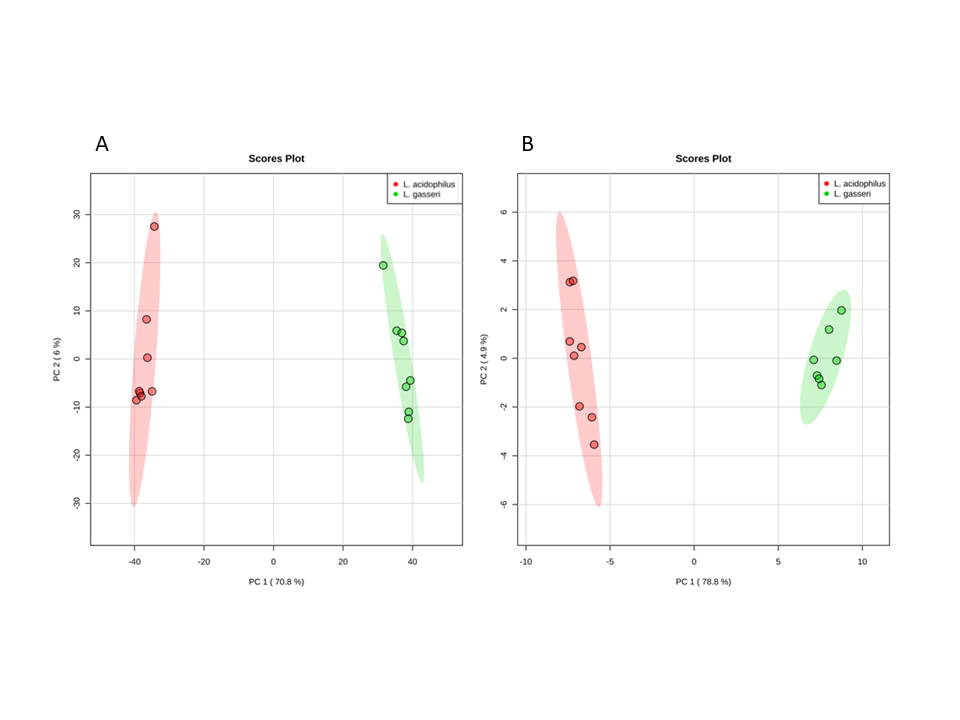

Supplement: S1 Fig — PCA depicts clear separation and analytical distinction between the global metabolomes (A) and lipidomes (B) of L. acidophilus and L. gasseri. In the metabolomics analysis, 76.8% of the variance is explained in 2 PCs, mostly accounted for by PC1, and 89.0% explained in 5 PCs. In the lipidomics analysis 83.7% of the variance is explained in 2 PCs, mostly accounted for by PC1, and 94.0% explained in 5 PCs. (TIF) [file pone.0222393.s001.tif]
